# Genome-wide mapping of promoter-anchored interactions with close to single-enhancer resolution

**DOI:** 10.1186/s13059-015-0727-9

**Published:** 2015-08-03

**Authors:** Pelin Sahlén, Ilgar Abdullayev, Daniel Ramsköld, Liudmila Matskova, Nemanja Rilakovic, Britta Lötstedt, Thomas J. Albert, Joakim Lundeberg, Rickard Sandberg

**Affiliations:** KTH - Royal Institute of Technology, Science for Life Laboratory, School of Biotechnology, Solna, 171 65 Sweden; Ludwig Institute for Cancer Research, Stockholm, 171 77 Sweden; Department of Cell and Molecular Biology, Karolinska Institutet, Stockholm, 171 77 Sweden; Roche Nimblegen Inc., R&D, Madison, WI 53719 USA

## Abstract

Although the locations of promoters and enhancers have been identified in several cell types, we still have limited information on their connectivity. We developed HiCap, which combines a 4-cutter restriction enzyme Hi-C with sequence capture of promoter regions. Applying the method to mouse embryonic stem cells, we identified promoter-anchored interactions involving 15,905 promoters and 71,984 distal regions. The distal regions were enriched for enhancer marks and transcription, and had a mean fragment size of only 699 bp — close to single-enhancer resolution. High-resolution maps of promoter-anchored interactions with HiCap will be important for detailed characterizations of chromatin interaction landscapes.

**Electronic supplementary material:**

The online version of this article (doi:10.1186/s13059-015-0727-9) contains supplementary material, which is available to authorized users.

## Background

Enhancers are *cis*-acting DNA elements, essential for the regulation of transcription at nearby genes [1]. Although numerous methods exist for the genome-wide mapping of enhancers, e.g., STARR-seq [2] and ChIP-seq for transcription factors (TFs) [3], co-factors [4], chromatin modifications [5], and DNA hypersensitive sites [6], it is still challenging to globally identify the promoters regulated by each enhancer. Since enhancer regulation is mediated via genome looping, which physically brings distant regions into close proximity [7], selected promoter–enhancer interactions can be investigated using chromatin conformation capture (3C) [8]. Using a specific region as bait (e.g., a promoter), chromosome conformation capture coupled with sequencing (4C) [9, 10] can be used to map genome-wide interactions with the bait region at high sensitivity and resolution. Genome-wide chromatin interaction was first studied de novo with the development of Hi-C [11] that selected for ligated fragments without using any particular regions as baits. This method was successfully used to identify topological domains and higher-order chromatin interaction patterns [12]; however, its 5–20 kb resolution prevents mapping of individual promoter–enhancer interactions [13], and improvement in resolution scales with the square of the sequence depth. Chromatin interaction analysis by paired-end tag sequencing (ChIA-PET) was developed to enrich for long-range interactions involving specific DNA binding factors [14] or actively transcribed regions [15]. Although ChIA-PET has higher resolution than Hi-C, the dependence on specific proteins for the immunoprecipitation reduces analyses to specific enhancers or actively transcribed genes. In parallel to the development of ChIA-PET, capture probes have been designed to hundreds of specific chromatin regions to improve 3C resolution, in a method called Capture-C [16]. Recently, genome-wide interaction maps have been generated by combining Hi-C with capture probes targeting all promoters. They employed six-cutter restriction enzymes and obtained detailed chromatin maps at an average resolution of 3.4 kb [17, 18]. Another recent study [19] combined Hi-C with capture probes against 998 long non-coding RNA genes. Using DNase I instead of a restriction enzyme, they obtained smaller fragment size and the identification of hundreds of interactions at 1 kb resolution.

In this study we have developed HiCap, enabling the generation of genome-wide maps of promoter-anchored chromatin interactions with close to single-enhancer resolution. A strong enrichment was observed for interactions with distal regions harboring enhancer-associated marks and those were frequently transcribed. Additionally, we demonstrate that HiCap interactions contain gene regulatory information through integrative analyses of TF over-expression and genome-wide binding (ChIP-seq) data.

## Results

### Development of HiCap

To identify genome-wide interactions anchored on promoters, we started by experimenting with 3C and Hi-C procedures together with sequence capture of promoter regions. We constructed capture probes that targeted restriction fragments containing the annotated promoters for essentially all mouse genes (31,127 promoters in 16,696 unique genes) and additional control regions in intergenic regions and exons (*n* = 184) (Table S1 in Additional file 1). We first investigated extensions of the Capture-C procedure to genome-wide level (by coupling 3C with sequence capture), but observed that Capture-C strongly enriched for un-ligated fragments, producing few read pairs with informative (>1 kb apart) junctions (Fig. S1 in Additional file 2). Instead, we based HiCap on modified Hi-C followed by a sequence capture of promoter-containing fragments (Fig. S2 in Additional file 2). While published Capture-C libraries [16] contain 1.3–2.5 % read pairs with informative connectivity information (i.e., a 1 kb to 10 Mb distance between the read pair), the HiCap libraries had much higher content (26–46 %) of such read pairs (Fig. S1 in Additional file 2). We calculated library complexity, i.e., the number of unique DNA fragments, using Preseq [20], which extrapolates from read duplicate frequency, and found that the HiCap libraries also had higher complexity than Capture-C libraries per input amounts of cells (7.7-fold difference, *P* = 0.009, *t*-test; Table S2 in Additional file 1). To obtain high-resolution interactions, we performed the Hi-C step of HiCap using a 4-cutter (MboI), which has a theoretical mean fragment size of only 422 bp in the mouse genome (Fig. S3 in Additional file 2). We generated two HiCap libraries (biological replicates) from mouse embryonic stem cells (mESCs) and sequenced the libraries from both ends (2 × 100 bp) to a depth of 200–300 million read pairs. HiCap reads were mapped independently and read pairs were discarded if they mapped within 1 kb of each other (to remove self-ligated fragments) or were deemed invalid using a computational procedure developed for analyses of Hi-C read data [21]. We calculated the efficiency of the restriction enzyme MboI as 71 %, using quantitative PCR (Table S3 in Additional file 1). The promoter capture efficiency, i.e., the percentage of aligned reads mapping on targeted promoter regions (which constituted 0.4 % of the genome), was estimated to be 18–44 % (Table S4 in Additional file 1), corresponding to 45–110-fold read enrichment at promoters.

### High-resolution mapping of promoter-anchored interactions

**Fig. 1:**
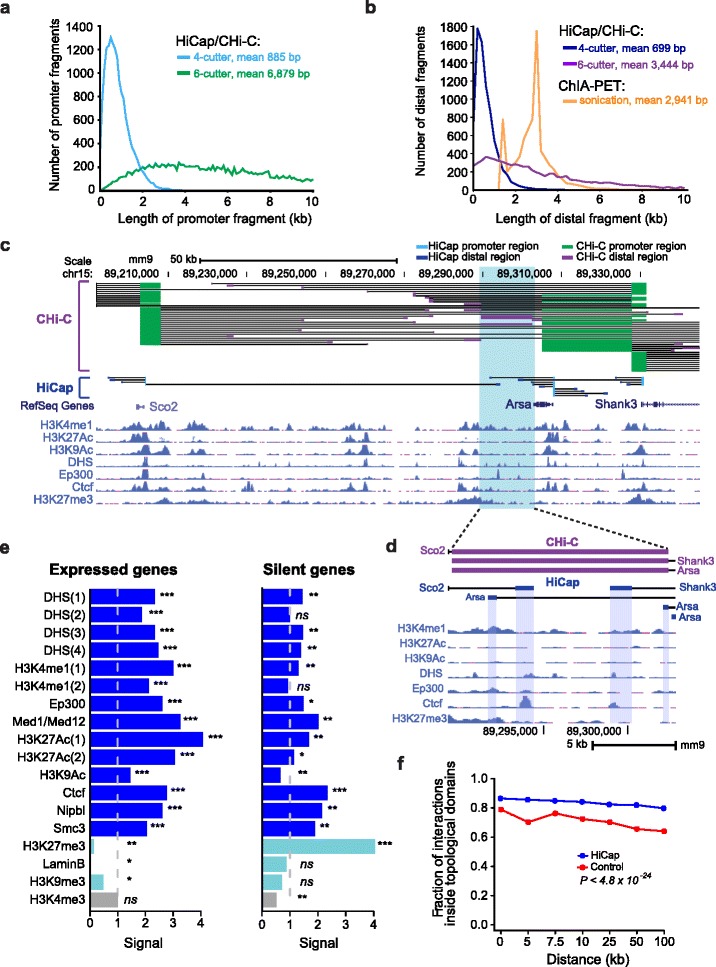
Genome-wide promoter-enhancer mapping with HiCap. **a** Length distribution of sequence-captured promoter regions in mESCs by HiCap and CHi-C based on either a 4-cutter (*turquoise*) or a 6-cutter (*green*). **b** Length distribution of distal regions identified by HiCap/CHi-C 4-cutter (*blue*), 6-cutter (*purple*) and ChIA-PET (*orange*) in mESCs. **c** Snapshot of observed promoter-anchored interactions for three genes (*Sco2*, *Arsa* and *Shank3*) in HiCap and CHi-C data, overlaid with genome-wide enhancer and chromatin marks. **d** Detailed zoom-in on one distal region identified by HiCap/CHi-C (6-cutter), which contains multiple smaller distal regions identified by HiCap/CHi-C (4-cutter). Promoters and distal regions are color coded as in (**a, b**). Gene names indicate which gene the distal region is interacting with. In both cases, these distal regions are interacting with same genes (*Sco2*, *Arsa* and *Shank3*). **e** Signal (observed overlap divided by expected) between HiCap promoter-anchored interactions mapping to distal regions and published genome-wide enhancers (*blue*), chromatin marks for silent genes (*turquoise*) and promoter marks (*gray*) in mESCs. HiCap distal regions were classified into expressed [>3 RPKM (reads per kilobase of gene model and million uniquely mapped reads)] and silent (≤0.3 RPKM) by the expression of their target genes. Significant (*χ* ^2^ test) comparisons are indicated with asterisks: *P < 0.05, **P < 0.001; ***P < 10^−10^; *ns* not significant. **f** Fraction of observed HiCap interactions contained within topologically associating domains (TADs), as a function of the interaction distance and compared with expected

To identify genome-wide promoter-anchored interactions, i.e., interactions with one read mapping to a targeted promoter region and its pair mapping elsewhere in the genome, we required the interactions to be supported with three or more reads in both biological replicates (Fig. S4 in Additional file 2). This resulted in the identification of 94,943 interactions involving 15,905 promoters (corresponding to 12,874 genes) and 71,985 distal regions. (Tables S5 and S6 in Additional file 1). Hereafter, we refer to the genomic regions observed to interact with one or more promoters as distal regions. First, we determined to what extent the resolution to call promoter-anchored interactions was improved with HiCap over previous methods that were based on either sonication (ChIA-PET) or a 6-cutter (CHi-C). To this end, we compared the lengths of our promoter and distal regions with those identified in published ChIA-PET interaction data generated with RNA polymerase II immunoprecipitation [15, 22] and Capture-Hi-C data in mESCs [17]. The 4-cutter resulted in much higher resolution for both the promoter and distal regions. The promoter fragments used for sequence capture were significantly shorter (mean 885 bp) compared with 6879 bp in a recent study [17] (Fig. 1a), which increased our ability to identify promoter-anchored interactions with proximal enhancers, e.g., those residing within the larger HindIII fragments. For distal regions, ChIA-PET and Capture-Hi-C data had a mean fragment length of 3789 and 3444 bp, respectively, whereas HiCap had significantly (*P* < 2.2 × 10^−16^, *χ*^2^ test) shorter fragments (mean 699 bp) (Fig. 1b), an adequate resolution to start mapping individual enhancers. However, it is important to note that ChIA-PET is designed to identify interactions mediated by protein complexes and the difference in resolution is thus compensated for by the specificity of the interaction information obtained. Visualizing the promoter-anchored interactions obtained for three genes (*Sco2*, *Arsa* and *Shank3*) in mESCs with our 4-cutter strategy and a recent 6-cutter study [17] illustrated the benefits of increased fragment resolution (Fig. 1c). HiCap could distinguish between four promoter-anchored interactions (coming from three different genes) targeting four closely located regions (Fig. 1d) that were indistinguishable using a 6-cutter strategy. We observed hundreds of similar examples in which multiple HiCap distal regions were found within HindIII fragments used in CHi-C, as expected (Fig. S5 in Additional file 2). Likewise, using 6-cutter strategies for promoter-anchored interactions might be complementary as they, by design, identify longer-range interactions.

### HiCap interactions are enriched for regions with enhancer features

In order to characterize the high-resolution promoter-anchored HiCap interactions, we investigated to what extent the HiCap distal regions overlapped with those enriched with enhancer-associated features from ChIP-seq and DNase hypersensitivity experiments (Table S7 in Additional file 1), henceforth referred to as “putative enhancers”. We excluded promoter–promoter interactions for these analyses. Overall, 64 % of the promoter-anchored HiCap distal regions overlapped putative enhancers, and we next assessed the enrichment of specific enhancer features in the distal regions as the ratio of observed to expected overlap. Expected overlaps were computed through randomly sampling fragments from annotated promoters using the observed distance distributions of HiCap interactions (preserving the non-random locations of promoters and enhancers in our background model). We found that HiCap distal regions interacting with promoters of expressed genes [RPKM (reads per kilobase of gene model and million uniquely mapped reads) >3] were significantly (*P* < 10^−21^, Chi-square (*χ*^2^) test) enriched for putative enhancers (Fig. 1e) carrying active marks, and significantly depleted for chromatin regions carrying repressive marks such as H3K27me3, Lamin B1 and H3K9me3 (*P* = 9.6 × 10^−8^, *P* = 9.2 × 10^−8^ and *P* = 0.014, respectively, *χ*^2^ test). Moreover, these distal regions were not enriched (*P* = 0.86, *χ*^2^ test) for promoter-associated H3K4me3 marks. In contrast, distal regions connected to promoters of transcriptionally silent genes were strongly enriched for the repressive chromatin mark H3K27me3 (*P* = 3.3 × 10^−13^, *χ*^2^ test; Fig. 1e). Furthermore, regions interacting with negative controls were significantly depleted for enhancer-associated chromatin marks and showed a significant enrichment for repressive chromatin marks (Fig. S6 in Additional file 2). Overall, these results demonstrate that the promoter-anchored interacting regions were highly enriched for regions with enhancer-associated chromatin marks or protein complex binding. As expected, we also observed that most HiCap interactions were contained within the same topologically associating domains (TADs; *P* < 4.8 × 10^−24^, *χ*^2^ test) and they were also depleted outside TADs (*P* < 3.2 × 10^−171^, *χ*^2^ test) (Fig. 1f; Fig. S7 in Additional file 2).

### Expression of enhancer RNA from mapped distal regions

**Fig. 2:**
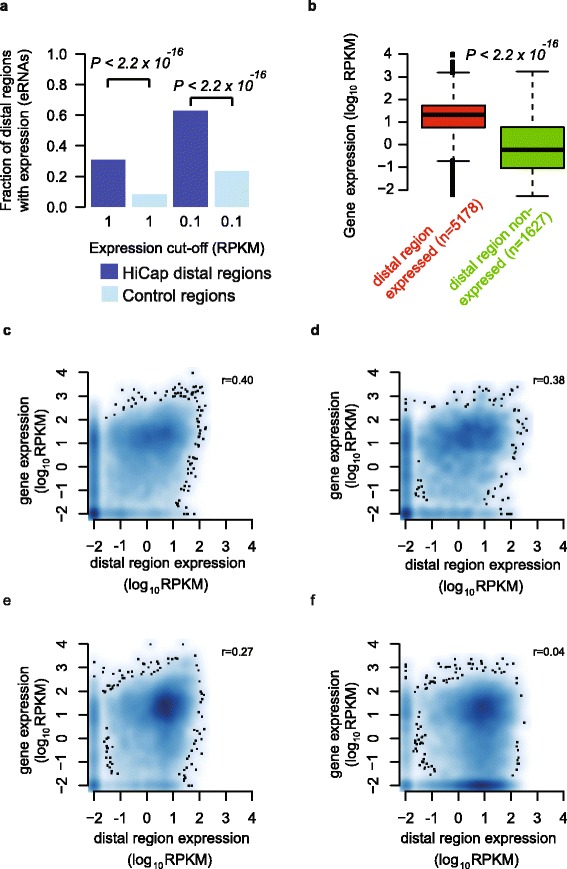
Expression of HiCap-identified distal regions and their correlations with target gene expression. **a** Fraction of HiCap distal regions with expression above 0.1 or 1 RPKM compared with random regions sampled within the same distances from promoters as observed interactions. **b** Boxplot comparing the expression of genes connected to either highly expressed (>10 RPKM) or non-expressed (≤0.3 RPKM) distal regions; number of genes is given in parentheses. **c** Spearman correlation of the expression of HiCap-mapped distal regions and their closest HiCap connected target genes. **d** Spearman correlation of the expression of HiCap-mapped distal regions and the closest gene without HiCap interaction. **e** Spearman correlation of expression of HiCap distal regions and non-closest HiCap connected target genes. **f** Spearman correlation of expression levels of HiCap distal regions and the expression of randomly connected non-closest genes on the same chromosome. When multiple distal regions are connected to the same gene, the RPKM sum was used for analyses in (**b**–**f**)

We observed that HiCap distal regions were often expressed; e.g., 30 % had expression above 1 RPKM (Fig. 2a). Moreover, distal regions were significantly more often expressed than random intergenic regions within the same distances from promoters (*P <* 2.2 × 10^−16^, Wilcoxon rank sum test; Fig. 2a). Importantly, HiCap distal regions connected to active promoters had significantly higher expression levels than those connected to silent promoters (*P =* 6 × 10^−45^, Wilcoxon rank sum test; Fig. 2b). We also observed that the expression levels of distal regions and the connected gene with a HiCap interaction mapped to its promoter were more highly correlated (*P =* 0.001, permutation test; Fig. 2c) than the closest genes of distal regions without a HiCap connection (Fig. 2d). Furthermore, the expression of HiCap distal regions connected to non-closest genes also showed significantly higher correlation (*P =* 0.001, permutation test; Fig. 2e) than random non-closest genes on the same chromosome at the same distance apart (Fig. 2f). In conclusion, both enhancer-associated chromatin marks and enhancer RNA expression were found at HiCap distal regions in support of HiCap enrichment for promoter-anchored interactions involving enhancer regions.

### HiCap interactions predict differentially expressed genes upon TF over-expression

**Fig. 3:**
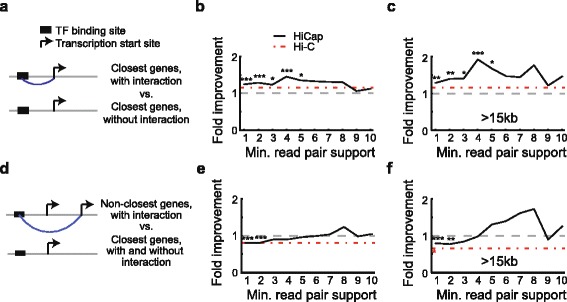
Validation of promoter–enhancer interactions by gene expression perturbation. Functional tests evaluating the predictive capabilities of HiCap- or Hi-C-mapped promoter–enhancer interactions. Transcription factor binding sites (TFBSs) were associated with promoters using either HiCap or Hi-C [12] interactions and compared with the set of genes closest to each TFBS. Gene sets were compared with upregulated genes from TF over-expression experiments, and fold improvement was computed based on the fraction of upregulated genes with HiCap (*black*) or Hi-C (*red*) interaction support over comparison gene sets (i.e., closest genes). **a** Comparison of only closest genes (to mapped TFBSs ) with interaction support with closest genes without interaction support. **b** Fold improvement in the fraction of upregulated genes among the closest genes with interaction support divided by the fraction of closest genes lacking interaction support. Significant (Fisher’s exact test) comparisons are indicated with asterisks: *P < 0.05, **P < 0.01; ***P < 0.001. **c** Like (**b**) but using a minimum 15-kb interaction distance. **d** Comparison between only non-closest genes (to mapped TFBSs ) with interaction support and closest genes (irrespective of interaction support). **e, f** Fold improvement in the fraction of upregulated genes among genes identified based on interactions with the set of closest genes. Details as in (**b-c**). Promoter–promoter interactions were excluded in all analyses for this figure

Although several studies have mapped genome-wide chromatin interactions [11–15], it remains to be determined whether the interactions are sufficiently enriched for bona fide regulatory interactions to be predictive of gene expression levels, in particular in comparison with the current best practice, which is to link enhancers to their closest genes. To this end, we re-analyzed genome-wide binding locations of 15 different TFs in mESCs together with genome-wide differential expression analyses after TF overexpression to determine whether genes with HiCap interactions to putative enhancers were more often found upregulated. We first focused on the closest genes to mapped TF binding sites (Fig. 3a) and found that genes with HiCap interaction support for mapped TF binding sites were more often upregulated than those without HiCap interaction support (Fig. 3b). The higher enrichment was significant (*P* < 0.001, Fisher’s exact test) for HiCap interactions at several read thresholds (Fig. 3b). There was also a trend for published Hi-C interactions [12] to agree with the overexpression data.

To investigate the functional relevance of interactions between distal regions and non-closest genes they are connected to, we evaluated their enrichment for upregulated genes. HiCap interactions mapped to more distant (non-closest) genes had similar and sometimes even higher enrichment for upregulated genes than the set of closest genes (Fig. 3c, d). Linking distant genes using the Hi-C dataset, however, resulted in worse enrichment than the closest gene set. Passing this rather strict perturbation-based validation test (strict since the effect sizes were compared with the effects for closest genes which often are targets) gives confidence that HiCap interactions reflect TF and, by extension, enhancer action.

### Network analyses of HiCap interactions

**Fig. 4:**
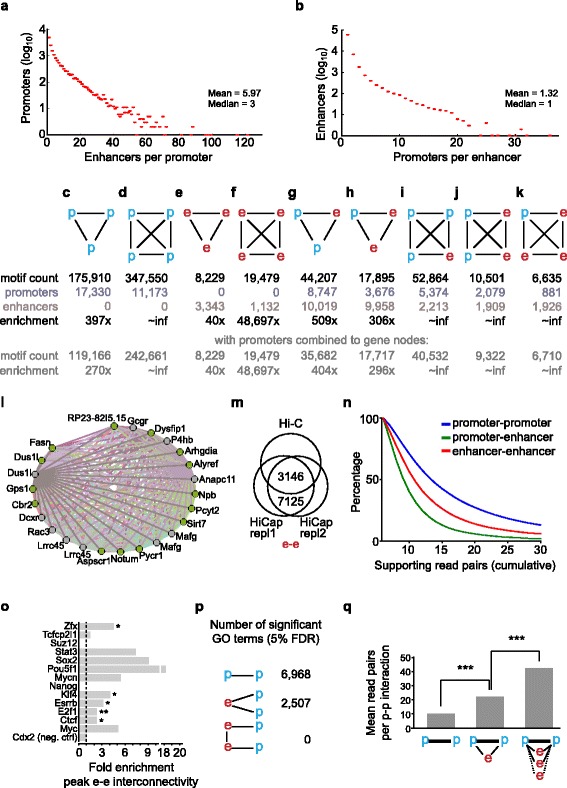
Motifs and interconnected clusters of promoters and enhancers. **a, b** Distributions of interaction for promoters (**a**) and enhancers (**b**). **c**–**k** Interaction motifs involving promoters (*p*) and/or enhancers (*e*) together with their occurrence among HiCap interactions and the numbers of unique promoters and enhancers within the motifs. **l** The most highly interconnected set of promoters, arranged by genomic coordinates and annotated with gene name. Promoters in *green* are all connected to each other whereas the ones in *grey* are missing some interactions. **m** Overlap between enhancer–enhancer interactions identified in HiCap and in-house Hi-C interactions. **n** Percentage of interactions supported with different read pair thresholds. **o** Mean degree for nodes (i.e., enhancers) in subnetworks of only enhancer nodes bound by the same TF or chromatin remodeling protein. Asterisks indicate significance (*P < 0.05, **P < 0.01, ***P < 0.001) computed against randomized enhancer node selections in the network. **p** Number of significant gene ontology (*GO*) categories enriched among gene pairs that share the same category, for genes connected by HiCap interaction motifs. *FDR* false discovery rate. **q** Mean read pair support for promoter pairs with increasing number of common enhancer interactions (0, 1 or ≥2)

Most distal regions interacted with only one promoter (1.32 promoters on average), whereas the promoters interacted with 5.97 distal regions on average, often within 1–100 kb and both degree distributions followed a power-law indicative of a robust network topology [23] (Fig. 4a, b). Since gene regulation in the nucleus has a spatial component [24, 25], we investigated whether global HiCap interactions could inform about general organization of regulatory interactions. We noted an apparent enrichment for interconnected clusters (cliques) of only promoters (Fig. 4c, d), only distal regions (Fig. 4e, f) as well as motifs involving both distal regions and promoters (Fig. 4g–k). The largest promoter cliques we found involved 19 promoters each, all involving a group of genes on chromosome 17 (Fig. 4l). Interactions involving two promoters were likely over-represented due to sequence capture. But we also detected high read support for interactions involving two distal regions, which surprised us considering these regions were not enriched for by sequence capture. We did rediscover those interactions in our 4-cutter Hi-C data (Fig. 4m; Fig. S8 in Additional file 2). Moreover, read support for interactions involving two distal regions was higher than for interactions between a promoter and a distal region (Fig. 4n), providing additional support for enhancer–enhancer interactions [12] and indicating that they are prevalent (Table S6 in Additional file 1). Further analyses of the interactions between distal regions revealed that they were more often bound by the same TF than what would be expected by chance, with significant enrichments for Zfx, Klf4, Essrb, E2f1 and Ctcf, and a trend towards enrichment for other factors (Fig. 4o). Interestingly, genes connected through promoter interactions or mutual interactions to the same distal regions were more often annotated to belong to the same gene ontology categories (Fig. 4p; Table S8 in Additional file 1), supporting the previous finding that such interactions could be involved in transcriptional coordination [15]. Finally, we observed that pairs of genes with interacting promoters had, on average, higher read pair support if they were additionally interacting with one or more distal regions (Fig. 4q).

## Discussion

We developed HiCap for the genome-wide identification of regulatory interactions. It was important to base the method on Hi-C, rather than 3C as in Capture-C, to obtain sufficient enrichment for ligated fragments. Using HiCap, we observed stronger enrichments for ChIP-seq inferred enhancers than with existing methods, likely due to incorporation of sequence capture targeting promoter sequences. Also, HiCap provides better sensitivity compared with Hi-C by fixing one interaction partner, thereby overcoming the need to quadruple the sequencing depth to double the sensitivity. Considering 4C as a reference method with the highest resolution to identify interactions at selected loci, HiCap had comparable reproducibility to other genome-wide methods (Fig. S9 in Additional file 2). It would be possible to use a broader target set such as the ~600,000 Fantom5 putative promoters [26] (8.7 % of which are within our promoter set), although it might not be possible to fit such numbers onto current oligo synthesis arrays. A recent study [27] introduced a combination of Hi-C with selected sequence capture of three long genomic regions containing disease-associated SNPs in gene deserts to identify several interactions in breast cancer. This illustrates the flexibility in combining Hi-C with sequence capture probes for disease-associated regions in detail [27] or genome-wide promoter interactions as performed in HiCap (this study) and Capture-Hi-C [17, 18]. Although increased resolution allowed HiCap to identify distal regions within CHi-C bait and distal regions, it is important to note that using a 6-cutter during the Hi-C step enabled CHi-C to identify longer range interactions compared with HiCap, which employs a 4-cutter during the Hi-C step (Fig. S10 in Additional file 2). Since HiCap relies on promoter capture, it may not be well-suited for studying organisms where the promoter regions are not well-annotated.

The promoter-anchored chromatin landscape did not just interact with distal regions. We observed extensive promoter–promoter interactions, but also more surprisingly abundant interactions between two or more distal regions. Despite our enrichment for promoter-anchored interactions, which selected against such distal to distal region interactions, we observed them with comparable read support to promoter-anchored distal interactions. This result supports an early Hi-C-based observation of putative enhancer–enhancer interactions in mESCs [13], and indicates that interconnected enhancer regions might be interesting to explore functionally. The distal regions seem to be enhancers, as they interact with promoters and are usually occupied by enhancer-associated TFs. Additionally, our analyses indicate that interacting pairs of distal elements are enriched for occupation by the same TF, which might help explain the formation or function of these interactions.

Although a large fraction of distal regions (65 %) were connected to the closest gene, HiCap identified thousands of long-range interactions. Importantly, we demonstrated that interactions between distal regions and more distant (non-closest) genes were as enriched for genes that became upregulated after TF over-expression as the set of closest genes. Although our improvement has a modest effect size, our results suggest that target genes from ChIP-seq experiments should contain both closest genes together with HiCap interactions involving genes further away from the TF binding location without diluting the signal. Similar incorporation of Hi-C interactions would dilute signal and should be avoided. TF perturbation tests, such as the one introduced in this study, will be important to assess predictive abilities of interactions identified in existing and novel methods. At present, it demonstrates that regulatory interactions are significantly captured with HiCap, but at the same time that predictive power is modest.

## Conclusions

We describe a new strategy for high-resolution mapping of genome-wide chromatin interactions anchored on promoters. In order for our resolution to match the sizes of promoters and enhancers, we shifted from using a 6-cutter restriction enzyme to instead using a 4-cutter. This resulted not only in higher resolution of promoters and distal regions, but also higher enrichment for enhancer features in our distal regions than has been reported in previous studies. Therefore, the methodology developed in this study will be important for high-resolution characterization of genome-wide interactions involving promoters and enhancers.

## Materials and methods

### Culturing of mESCs

mESCs (line R1) were obtained from Janet Rossant’s lab (Toronto, Canada). Cells were maintained on 0.1 % gelatin-coated dishes in Dulbecco's modified Eagle medium (DMEM) supplemented with 10 % fetal calf serum, 0.1 mM non-essential amino acids, 0.3 mg/ml L-glutamine, 1 mM pyruvate (Invitrogen), and 1000 U/ml murine leukemia inhibitory factor (Chemicon International ESGRO), and were kept in a 5 % CO_2_ atmosphere at 37 **°**C. The medium of undifferentiated cells was changed daily.

### Experimental procedure of HiCap

Hi-C was performed on mESCs as previously described [11], except for the following modifications. We generated replicate experiments from ~5 million mESCs that were cross-linked with 1 % formaldehyde for 10 min. Cells were lysed and nuclei were isolated. Isolated nuclei were digested with 4-cutter FastDigest MboI (Thermo Scientific, 1 μl/μg DNA) for 4 h at 37 **°**C. The ends of digested material were filled with biotinylated dATP, dGTP, dCTP and dTTP using Klenow fragments (Fermentas, 0.1 U per 1 μg DNA). Klenow was deactivated using 0.01 M EDTA at 75 **°**C for 15 min. Then the material was diluted to 3.5 ng/μl and ligated using T4 DNA Ligase (Promega). The crosslinking was reversed by adding Proteinase K and incubating overnight at 65 **°**C. The proteins were removed and DNA was purified using phenol-chloroform followed by ethanol precipitation. Biotinylated but unligated ends were removed using T4 DNA polymerase by incubating at 12 **°**C for 15 min. The material was fragmented to 300–600 bp by sonication. The fragment ends were repaired and A-tailed. Then the biotinylated fragments were bound to streptavidin beads and unbound fragments were washed away. Sequencing adapters were then ligated to the fragments bound to beads. The material was amplified for six to nine cycles while bound to beads to obtain sufficient amounts for sequence capture. Original biotinylated material was removed and the supernatant was hybridized to a sequence capture probe set according to the manufacturer’s instructions (Roche Nimblegen Inc.). Hybridized material was washed according to the manufacturer’s instructions and amplified with PCR for three to six cycles.

Hybridization of the probes to the Hi-C material was done exactly according to the manufacturer’s instructions (Roche Nimblegen Inc). Briefly, 1 μg of Hi-C material was mixed with 5 mg COT DNA, 1 μl of 1000 μM Universal Oligo, and 1 μl of 1000 μM Index Oligo and dried down in a vacuum concentrator on high heat (60 **°**C). Then, 7.5 μl of 2× hybridization buffer and 3 μl of hybridization component A [these components are included in the Nimblegen SeqCap EZ Hybridization and Wash Kit (catalog number 05 634 261 001)] were added to the dried down material, mixed well by vortexing for 10 s and centrifuged for 10 s. The mix was placed in a 95 **°**C heat block for 10 min to denature the DNA, and then centrifuged for 10 s at maximum speed. The mixture was then transferred to a 0.2 ml PCR tube containing 100 ng of the appropriate probe set (4.5 μl volume). The mixture was vortexed for 3 s and centrifuged for 10 s and placed in a thermocycler set at 47 **°**C for incubation for 64–72 h. The thermocycler’s heated lid was set to 57 **°**C.

After the incubation, the mixture was washed to eliminate unhybridized probes. Wash buffers (Stringent, I, II and III) and 100 μl of streptavidin beads were prepared for each hybridization according to the manufacturer’s instructions. The hybridization mix was mixed with 100 μl of streptavidin beads, further mixed by pipetting up and down 10 times and placed back in the thermocycler at 47 **°**C for 45 min. After the incubation, 100 μl of 1× wash buffer I heated to 47 **°**C was added to the mix and vortexed for 10 s. The contents of the tube were transferred to a 1.5 ml tube that was placed in a magnet to bind the beads. The liquid was removed and discarded once clear. Stringent wash buffer (200 μl, 1×) heated to 47 **°**C was added to the beads, pipetted up and down 10 times and incubated for 5 min at 47 **°**C. The mix was then placed in the magnet and liquid was removed once clear. The wash with 1× Stringent wash buffer was repeated once more. Then, 200 μl of 1× wash buffer I was added to the mixture and mixed by vortexing for 2 min; the beads were collected using the magnet and liquid was discarded once it was clear. The same steps were then repeated using 300 μl wash buffer II (except this time vortexing for 1 min) and 200 μl wash buffer III (except this time vortexing for 30 s). To elute the captured material from the beads, 50 μl of PCR-grade water was added to the beads and they were stored at −20 °C until further use.

The resulting DNA libraries were sequenced 100 bp from both ends (paired-end sequencing) on a HiSeq 2000 (Illumina Inc.). This is long enough to map to ~90 % of the genome [28], including, e.g., dead retrotransposon repeats [87 % mappability for long terminal repeats, 82 % for long interspersed elements (LINEs), 98 % for short interspersed elements (SINEs)], as annotated by RepeatMasker and using mappability files from MULTo [28]. We performed a number of alternative washing procedures to see if we could improve sequence capture efficiency. However, we find that the washing procedure recommended by the manufacturer performed the best. Table S9 in Additional file 1 summarizes the alternative washing procedures tried and corresponding sequence capture efficiency (percentage of reads that are mapped on the probe sequences).

### Mapping of sequence data

Paired-end sequences were aligned to the mouse genome (build mm9) through HiCUP [21] which used Bowtie [29] version 0.12.7 in single-end mode for the two ends separately, and with iterative trimming from the 3’ end for unaligned reads. Multi-mapping reads were discarded. Paired-end mapping is not suitable for HiCap libraries as the 100 bp on either end often contain the ligation point so that a paired-end mapper would soft trim that sequence end, effectively removing the pairing information. We therefore used custom scripts to pair the independently mapped sequence ends and we indexed each sequence pair to their corresponding MboI restriction fragment.

### Sequence capture probes

We designed sequence capture probes against mouse promoters compiled from multiple sources. RefSeq and Ensembl annotations were used together with transcription start sites from DBTSS (from 25 May 2010) and MPromDb (from 28 May 2010). There were in total 53,501 target sequences (targeting closest upstream and downstream MboI sites of each promoter and negative controls) and the probes covered 93.5 % of the target bases (11,293,801 bases). DBTSS is based on full-length mRNAs, and mostly corresponds to RefSeq and Ensembl. MPromDb is based on RNA polymerase II and H3K4me3 ChIP-seq data for different cell types, including ESCs. From annotated transcript start sites, we searched for the closest restriction cut sites (GATC) on each side, and chose the last 150 bp before the cut site as the captured regions. When restriction sites were <300 bp apart we chose the whole region between them. From these regions, Nimblegen designed the actual probe sequences. We also selected exonic and intergenic control regions which were included in the same probe selection pipeline.

### Calling of interactions

HiCUP software available at Babraham Bioinformatics [21] was used to filter out non-informative and unlikely pair combinations. Read pairs with the exact same mapping positions were discarded (to remove any potential effect from PCR duplicates) and pairs less than 1 kb apart were excluded. We only used pairs with at least one read mapping to probe regions. We counted the number of times each pair is observed for each set of probes belonging to promoters to derive interaction read support. We required at least three supporting read pairs in each biological replicate to call an interaction. We did not see any correlation between the number of restriction enzyme fragments closest to a transcription start site and interactions originating from that transcription start site (r^2^ = 0.065, Pearson correlation coefficient). G+C content of HiCap distal regions was slightly higher than the genome average (47 ± 6.7); 40–70 % for 99 % of HiCap distal regions. Promoter–promoter interactions were called similarly, but required that both ends of the paired reads aligned with probes belonging to promoters. We also mined the raw read pairs for interactions involving only distal regions. For this purpose we collected all distal regions from significant promoter–distal interactions and performed similar analyses for read pairs with both ends originating from a HiCap distal region. Following is a breakdown of called interactions and how they distribute over expressed and non-expressed genes. We detected at least one interaction for 73 % of the expressed genes (11,786 out of 16,241, RPKM >0.3) and for 48 % of genes with no detectable expression (6532 out of 13,584). Unsaturated sequencing could account for the fact that we did not detect any interaction for 27 % of the expressed genes. It is not surprising, however, that we did not detect any interaction for 52 % of genes with no expression as they might not be involved in distal interactions. There are also cases where only one of the alternative promoters of the same gene is involved in a distal interaction; therefore, it is fairer to assess the number of genes with interactions rather than the number of promoters.

### Analyses of overlap with enhancer ChIP-seq data

We downloaded enhancer regions inferred from different ChIP-seq experiments carried out in mESCs (Table S7 in Additional file 1). We sorted the mapped regions in each experiment to analyze only the top 5000 mapped regions from each experiment, in order to control for different signals and background levels in the different experiments. For Mediator data, we downloaded raw reads for Med1 (SRX022694 and SRX022695) and Med12 (SRX022692 and SRX022693) and aligned these to the mouse genome mm9. We performed peak calling using SISSRs version 1.4, and concatenated and sorted the peaks. ChIP-seq mapped regions were extended to 1000 bp if they were shorter (relevant only for Mediator bound regions). For analyses of HiCap overlap with putative enhancers, we computed the observed to the expected overlap. To calculate the percentage of HiCap promoter–enhancer interactions overlapping with at least one enhancer mark we simply overlapped HiCap enhancers with Chip-seq associated mESC enhancer features from Additional file 1: Table S7. The observed overlap was simply computed as the fraction of HiCap interactions that overlapped (by at least one nucleotide) with enhancer mapped regions. To compute the expected overlap we randomly sampled regions close to annotated transcription start sites, using the actual distance distribution of HiCap interactions. We found this procedure to better control for the non-random locations of genes and enhancers in the genome, whereas the computation of expected overlap based on a fully random model (the fraction of genomic fragments overlapping putative enhancers) rendered all tests significant.

### Comparison of HiCap and in-house Capture-C with published Capture-C

We performed Capture-C (3C coupled with sequence capture) using our custom promoter probes. We downloaded Capture-C raw reads from the Gene Expression Omnibys (GEO) database with sample IDs [GEO:GSM1156607] and [GEO:GSM1156608] (for Ter119^+^ cells) and [GEO:GSM1156609] (for mESCs).

### Expression level analyses in mESCs

We prepared a RNA-seq library for mESCs using the Illumina mRNA-seq protocol. The library was sequenced with an Illumina GAIIx at 50 bp read length in single-end mode (Fasteris, Switzerland). Reads were aligned to the mouse genome (mm9 assembly) and a comprehensive collection of splice junctions [30] using Bowtie (version 0.12.7). Expression levels were estimated as RPKM using Rpkmforgenes [31], where only uniquely mappable positions were included in the gene model length. Mappability was determined using MULTo [28] and gene models were based on RefSeq annotation downloaded from the UCSC genome browser on 31 July 2011.

### Functional test of HiCap interactions

To assess the power of HiCap interactions to predict differentially expressed genes after TF perturbation, we constructed the following test (with results presented in Fig. 3). TF binding data were downloaded [32] (Table S3 in Additional file 1) as well as expression data after TF overexpression [33] (Table S2 in Additional file 1). For each TF present in both datasets, we listed the closest gene to each midpoint of the binding region. We identified HiCap interactions connecting promoters to the restriction fragment containing the binding site midpoint and listed the genes of those promoters. For Fig. 3b, c, we compared the closest gene of peaks without HiCap interactions with the closest genes that also had a HiCap interaction. For Fig. 3e, f, we compared non-closest genes with HiCap support with the set of closest genes (irrespective of HiCap interactions). This procedure was performed also on Hi-C interactions. We compared the fraction of upregulated genes present within the gene sets and report the differences as fold improvements, by dividing the two numbers by one another. To explain the test in detail for Fig. 3d–f, we computed the number of closest genes, Nc, and the number of HiCap-connected genes, Nh. From the expression data we identified differentially expressed genes after each TF perturbation independently (false discovery rate ≤0.05 and fold change >1.5). Next, we computed the number of unique genes that were differentially expressed and also present in either the set of closest genes (Uc) or HiCap inferred (non-closest) genes (Uh). We summarized the enrichment as fold improvement [Uh/Nh]/[Uc/Nc] and calculated a *P* value using the *χ*2 test with Uh and Uc as observed and Nh/Nc as their expected ratio. For the compound test including all TFs, we summed all values of Nc, Nh, Uc, and Uh and performed the same tests.

### Visualization of interactions

We downloaded a significant “promoter–other” interaction table for CHi-C and selected 548,551 interactions based on their log observed/expected value [17]. We made a GFF file for HiCap and CHi-C interactions and uploaded it to the USCS Genome Browser. We overlaid interactions on selected tracks of enhancer features (while keeping their default minimum and maximum data range unchanged).

### Analysis of TADs

We downloaded TAD coordinates from a Hi-C study on mouse ESCs [12]. We then calculated the fraction of HiCap interactions completely contained within a TAD, spanning two or more TADs, or with one or both ends outside annotated TADs. We performed the same analysis on control region interactions that were calculated by randomizing the chromosomes while keeping the distance the same as in HiCap. The fraction of interactions was calculated as a function of the distance between promoters and distal elements. *P* values were calculated using the *χ*^2^ test on each paired fraction and the highest *P* value was reported.

### Analyses of enhancer RNA expression

We re-analyzed mapped GRO-seq data present in the GEO (GSM1186440 and GSM1186441 combined) [34] to determine expression levels for HiCap-inferred distal regions. For that we used HiCap distal regions that do not overlap (intergenic, 42 %) with any genes from the RefSeq annotation. In parallel, we generated random regions located within the same distances from promoters as HiCap distal regions, having the same average length as HiCap distal regions and not overlapping with genes from RefSeq annotation. We calculated expression levels (RPKM) for distal and random regions using the Rpkmforgenes [31]. P-values were computed using *χ*^2^ test based on the fraction of total regions from HiCap and random with expression above either 0.1 or 1.0 RPKM.

### Interaction motifs

We mined the HiCap interactions between promoters and enhancers to enumerate the occurrences of motifs (Fig. 4a–j). As a background model to calculate enrichment, we randomized promoter–promoter, enhancer–promoter and enhancer–enhancer interactions separately five times but keeping the degree distributions. Motifs which did not occur in the background were assigned “~inf” enrichment. To calculate *P* values in Fig. 4q, we grouped interactions by distance (1000–1999, 2000–3999, 4000–7999, etc. up to 64,000–127,999) and by the sum of the degree of the promoter nodes (2, 3, 4, etc. up to 20). We then performed a one-tailed Wilcoxon rank sum test for each group (for 0 versus 1 or 1 versus 2+ enhancers), and combined the *P* values by Stouffer's z-score method, to compute two-tailed *P* values. The *P* values were also significant (*P* < 10^−300^) without this consideration for distance and network degree.

### Gene ontology analyses of interconnected gene pairs

We tested if gene pairs connected through promoter–promoter, promoter–enhancer–promoter interactions more often shared annotated gene function. To this end, we used the gene ontology service DAVID [35]. First we calculated for each gene ontology term how many gene pairs were connected through one or more HiCap interactions in the patterns outlined in Fig. 3m for genes associated with that gene ontology term. Then we randomized (n = 1000) all HiCap interactions among all promoters and enhancers and repeated the same analyses above. We computed *P* values as the number of randomizations with at least as many pairs as the non-randomized, or one less (to account for selecting terms with at least one real pair associated with them). Due to the 1000 randomizations, the minimum possible *P* value was 0.001. *P* values were then adjusted to false discovery rates using the Benjamini–Hochberg method.

### Data access

Raw sequence reads have been submitted to the NCBI Sequence Read Archive [36] under [SRA:SRP045579] and [SRA:SRP045580], and processed gene expression values and interaction files have been submitted to the NCBI GEO [37] under accession number [GEO:GSE60495].

## Additional files

Additional file 1:
**Supplementary tables.** (ZIP 20649 kb) Additional file 2:
**Supplementary figures.** (PDF 1269 kb)

## Abbreviations

3C: chromatin conformation capture
4C: chromosome conformation capture coupled with sequencing
bp: base pair
ChIA-PET: chromatin interaction analysis by paired-end tag sequencing
ChIP-seq: chromatin immunoprecipitation followed by high-throughput DNA sequencing
GEO: Gene Expression Omnibus
mESC: mouse embryonic stem cell
RPKM: reads per kilobase of gene model and million uniquely mapped reads
TAD: topologically associating domain
TF: transcription factor
