# Supplementary material for: Genome-wide mapping of promoter-anchored interactions with close to single-enhancer resolution
Source: Genome Biol. 2015 Aug 3;16(1):156. doi: 10.1186/s13059-015-0727-9 (PMC4557751; doi:10.1186/s13059-015-0727-9)
Supplement: Additional file 2: — Supplementary figures. (PDF 1269 kb) [file 13059_2015_727_MOESM2_ESM.pdf]

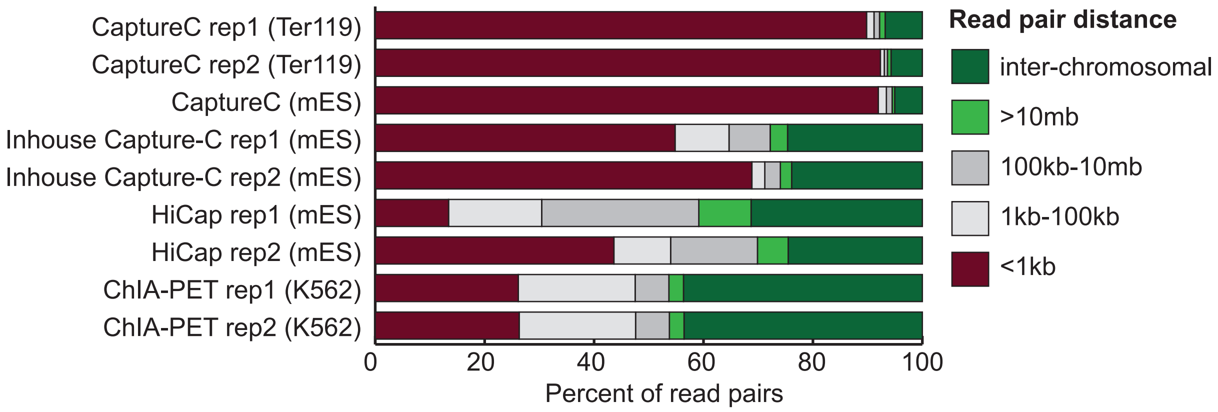

Supplementary Figure 1: Percentage of read pair distances compared between Capture-C (Ter119+ and mESC), HiCap, our inhouse Capture-C (mESC) and ChIA-PET (K562) replicates.

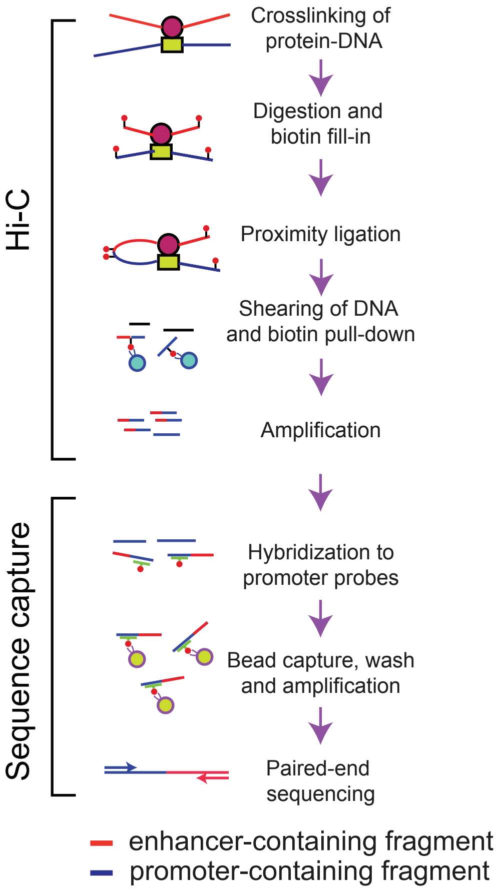

Supplementary Figure 2: Schematic illustration of the HiCap methodology

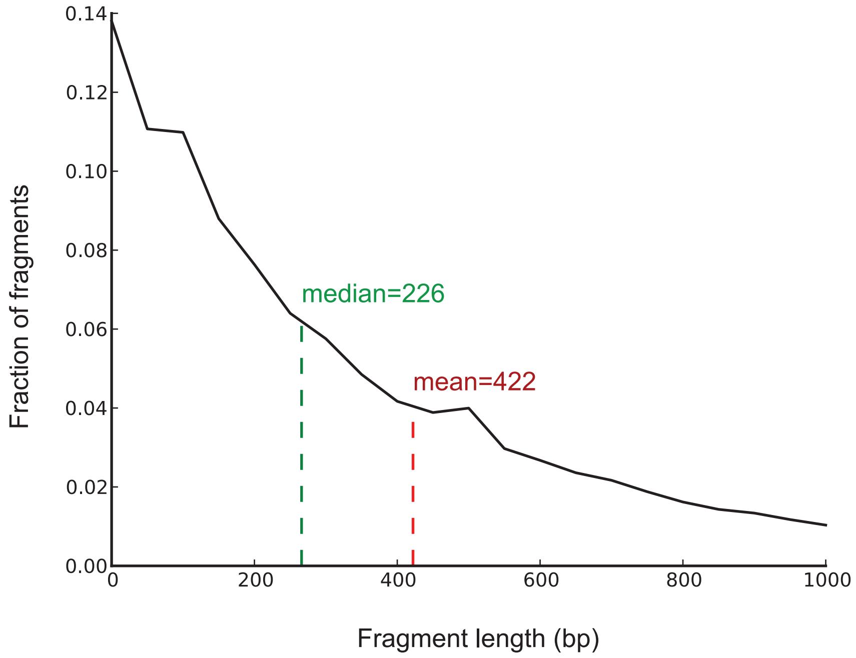

Supplementary Figure 3. Length distribution of MboI cut mouse genome.

We generated *in silico* a *MboI* fragmented mouse genome (mm9) to illustrate its theoretical fragment size distribution, together with mean and median sizes.

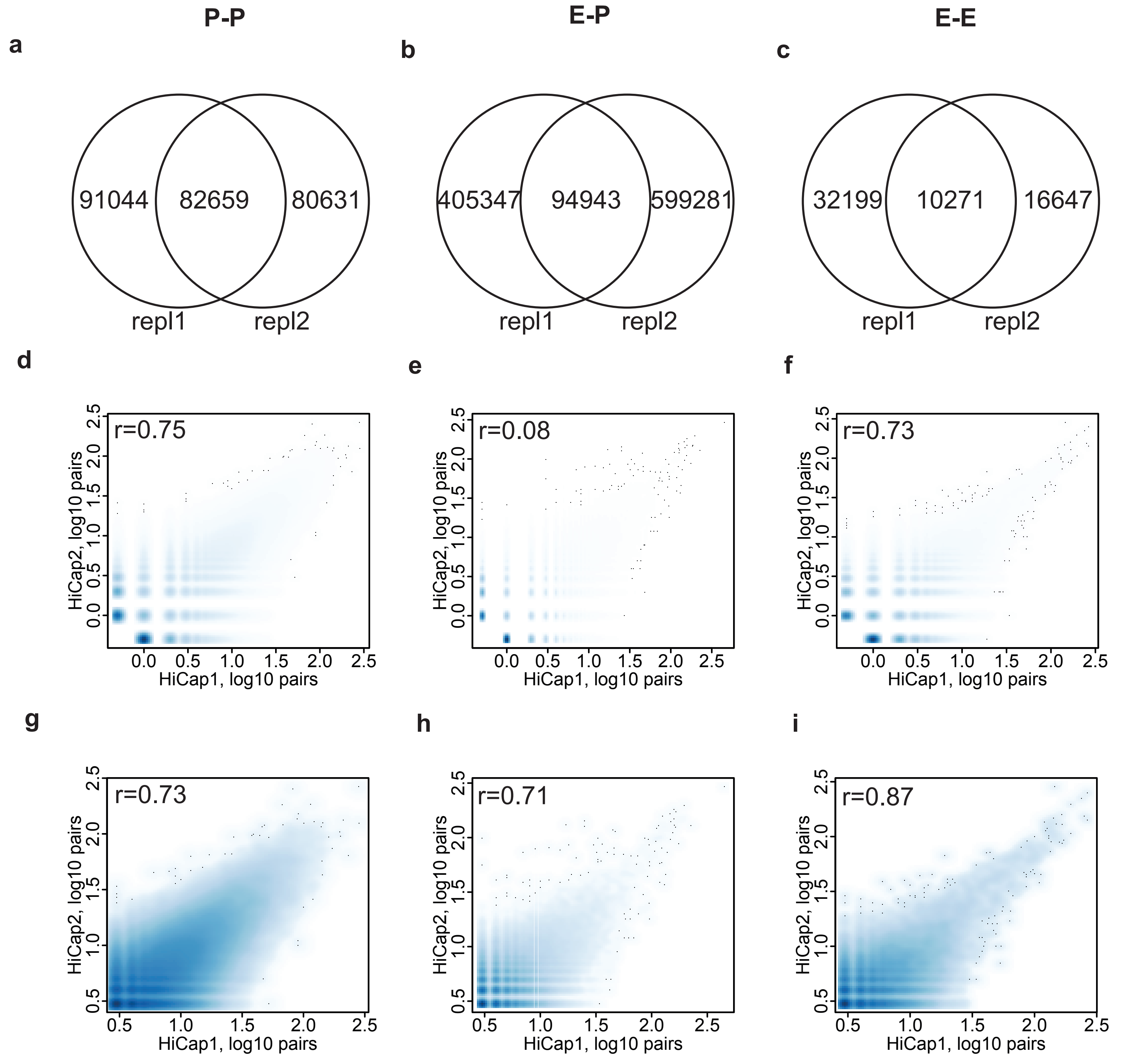

Supplementary Figure 4. Comparison of interactions identified in the two HiCap libraries. (a-c) Venn diagrams comparison of the significant interactions (at read threshold 3) identified in the two biological replicate experiments in mouse embryonic stem cells. Although the overlap is highly significant, many interactions were only identified in a single replicate. (d-f) Scatter plots of read pair support for promoter-promoter (d), promoter-enhancer (e) and enhancer-enhancer (f) interactions, without any threshold. r shows Pearson correlation. (g-i) Same as d-f, but only for significant interactions identified in the two biological replicates.

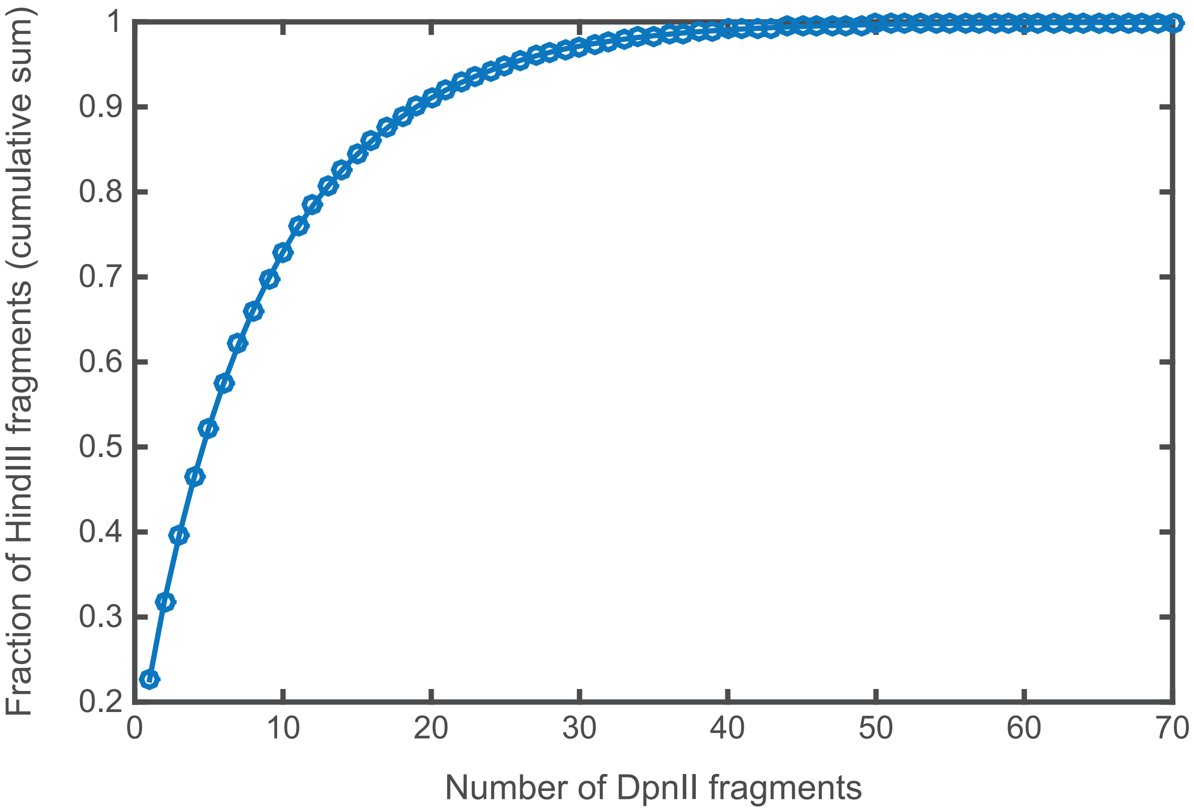

Supplementary Figure 5: Distribution of DpnII fragments found in HindIII fragments. Mouse genome mm9 was in silico digested into DpnII and HindIII fragments using restriction sites GATC and AAGCTT respectively. Number of DpnII fragments in HindIII fragments was calculated by summing the fraction of overlap of each DpnII site with HindIII fragment.

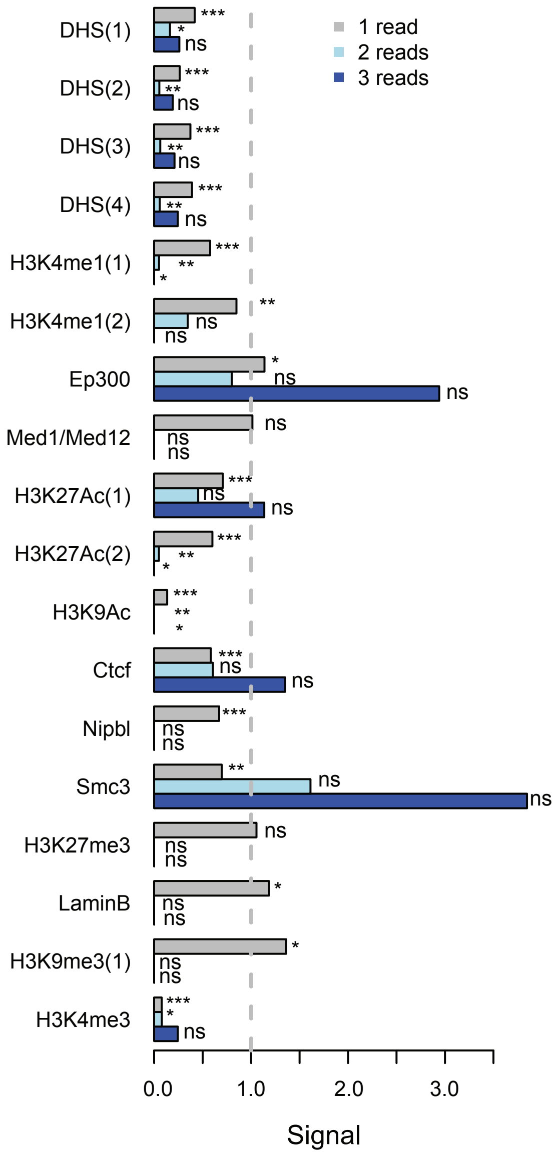

Supplementary Figure 6:
Enrichment of HiCap negative control regions for enhancer-associated marks

Signal enrichment of negative control regions for Chip-seq identified enhancer marks by increasing the number of read support identified in both biological replicates. Legend indicates the number of minimum read pairs supporting interactions. Significant (χ^2 test) comparisons are indicated with stars as defined below:
*P < 0.05, **P < 0.001; ***P < 10^{-10}

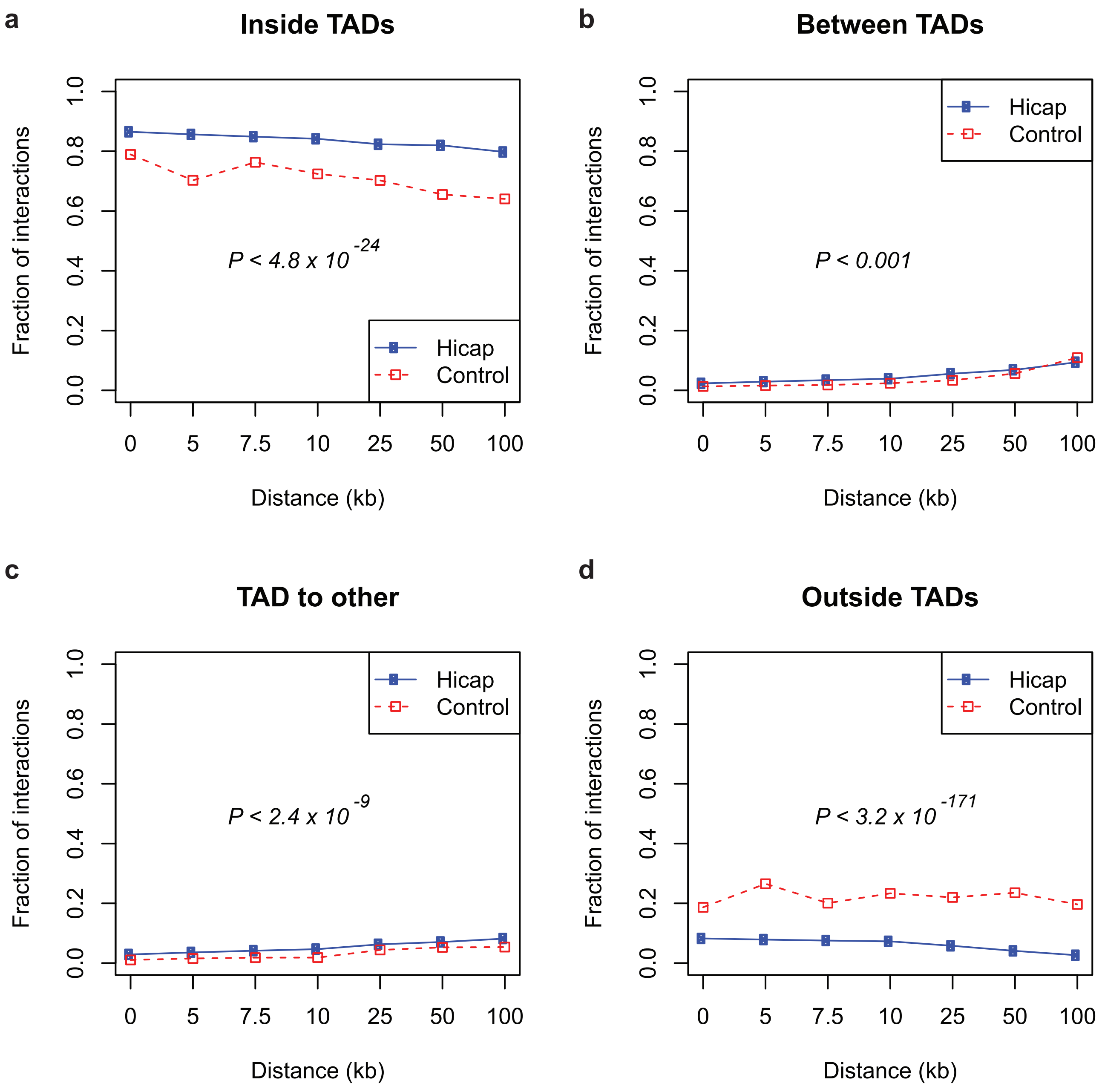

Supplementary Figure 7 Location of HiCap interactions with respect to topological associated domains (TADs). Fraction of HiCap and control interactions where (**a**) both promoter and distal element found in the same TAD, (**b**) both found in separate TADs, (**c**) either one found in TAD and (**d**) where none of them were found in TADs. Control interactions are calculated by randomizing the chromosomes while keeping the distance the same as in HiCap. Fraction of interactions are calculated as a function of the distance between promoters and distal elements. P values were calculated using Chi-square test. TADs were downloaded from Dixon JR, et al, 2012.

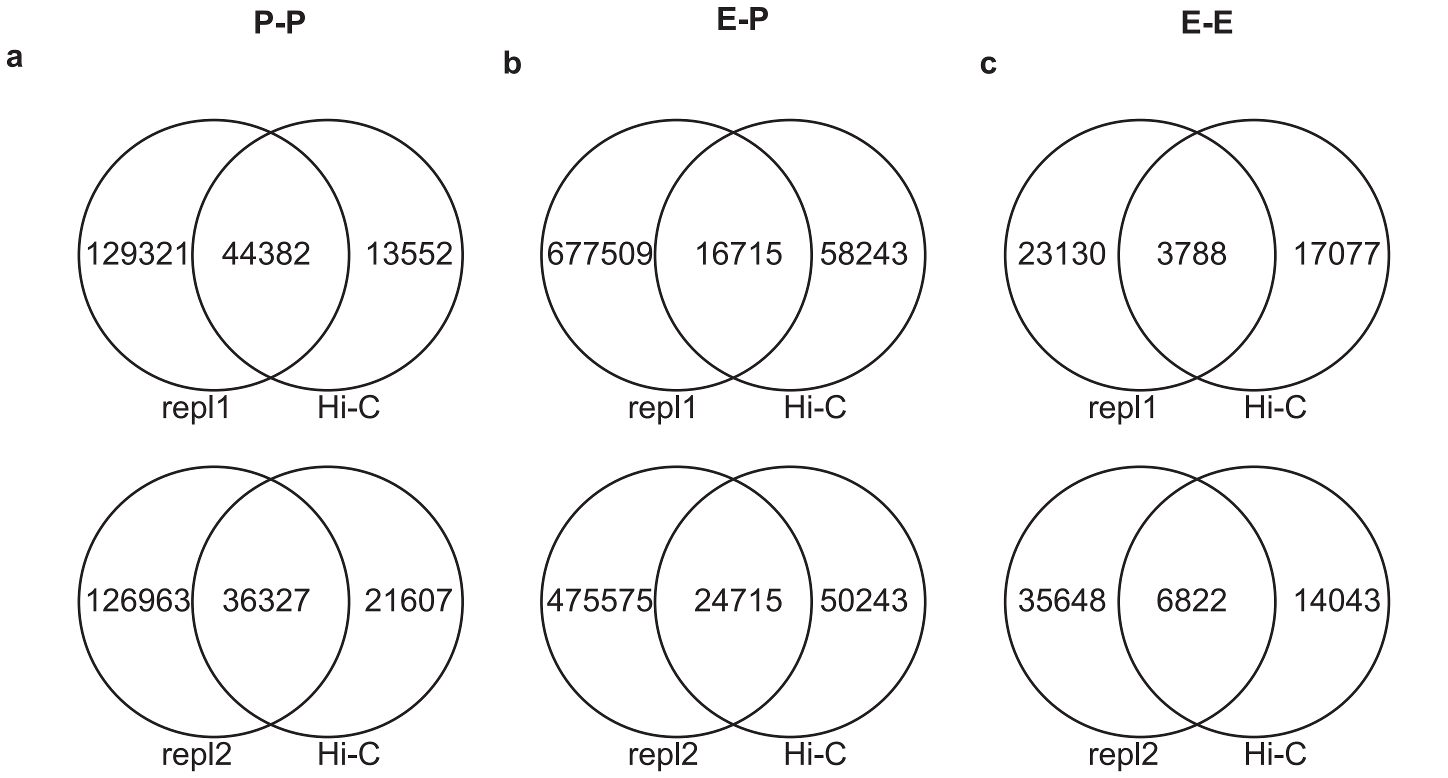

Supplementary Figure 8. Comparison of interactions identified in the HiCap and Hi-C libraries. (a-c) Venn diagrams of the interactions identified in the two biological replicate experiments, with interactions from our Hi-C data (at read threshold 3), for promoter-promoter (a), promoter-enhancer (b) and enhancer-enhancer (c) interactions.

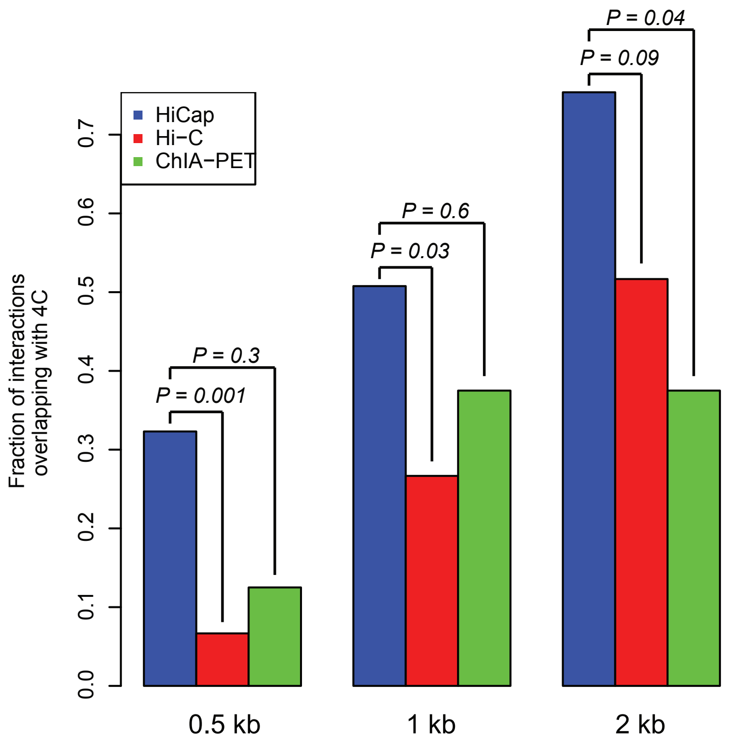

Supplementary Figure 9: Comparison of interaction reproducibility between genome-wide methods with respect to 4C method.

Barplot showing the fraction of interactions from genome-wide methods such as HiCap, Hi-C and ChIA-PET that overlap with interactions from high-sensitivity method such as 4C. We downloaded 11 mESC 4C datasets of interactions from 11 genes (*1700067P10Rik*, *Dppa3*, *Hoxa10, Maoa*, *Nfia, Pcdhb19, Pou5f1, Prss22, Rhbdd1*, *Tbx5*, *Vegfc* and *Zfp42*) from Gene Expression Omnibus series GSE50029. We compared the interactions of the same genes from HiCap, Hi-C and ChIA-PET to the corresponding gene interactions reported by 4C. In order to see assess the extent of overlap, 4C regions were extened 0.5 kb, 1 kb or 2 kb. Chi-square test was applied to calculate the p values.

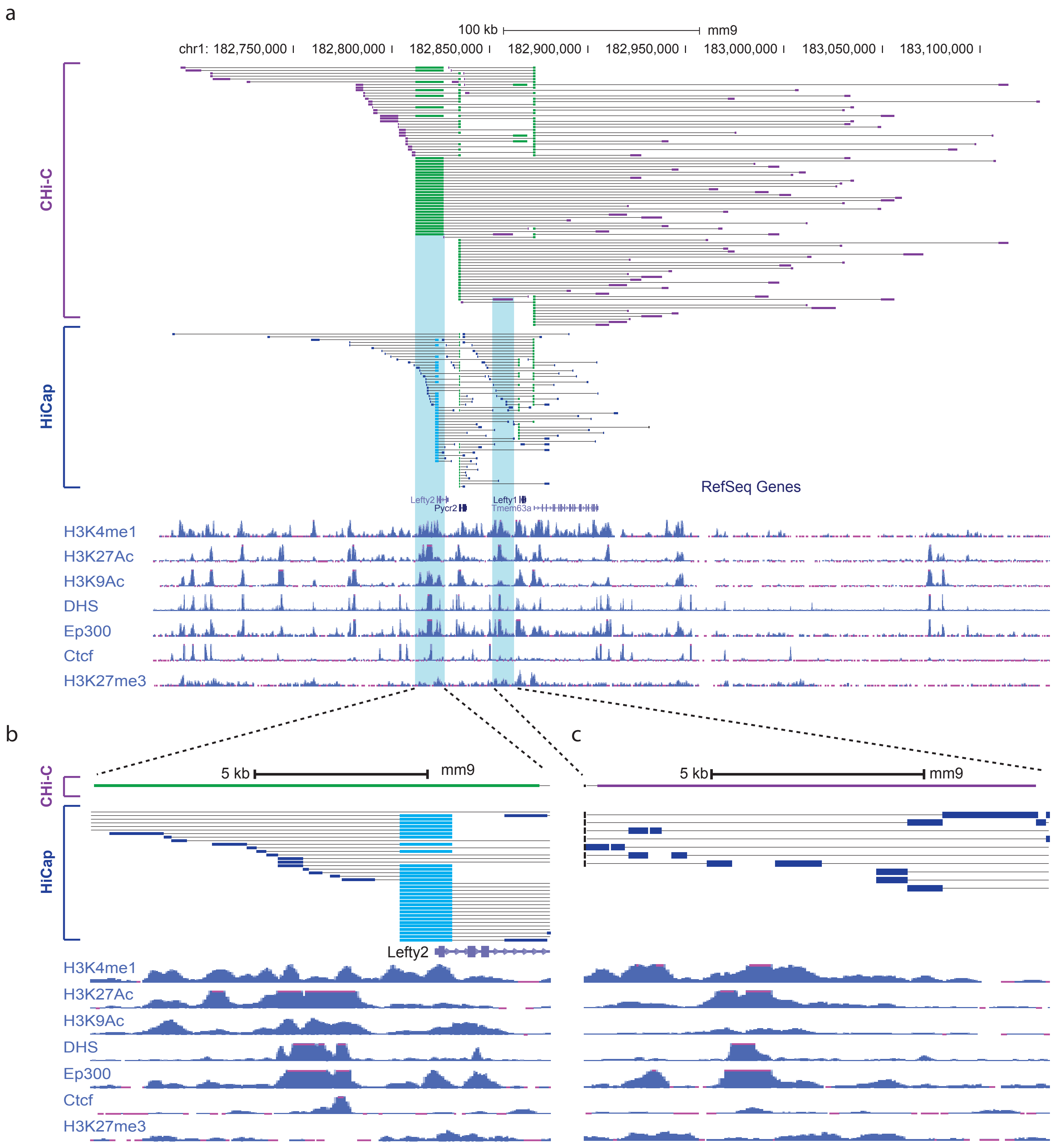

Supplementary Figure 10: Snapshot of interactions from CHi-C and HiCap.
a) CHi-C and HiCap interactions from 4 genes (*Lefty1, Lefty2, Pycr2 and Tmem63a*) overlayed on enhancer-associated marks. Baited regions (green boxes) and non-baited regions (black boxes) illustrates promoter and distal regions respectively. b) Zooming into the interaction window containing CHi-C bait region for *Lefty2* gene. c) Zooming into the interaction window containing CHi-C non-baited region (distal element) interacting with *Pycr2* gene. The interactions where both baited and non-baited regions located outside the zoomed window are not shown in (b) and (c).
